# Constructing agri-food for finance: startups, venture capital and food future imaginaries

**DOI:** 10.1007/s10460-022-10383-6

**Published:** 2022-10-31

**Authors:** Sarah Ruth Sippel, Moritz Dolinga

**Affiliations:** 1 Westfälische-Wilhems-Universität Münster, Institute of Geography, Heisenbergstr. 2, 48149 Münster, Germany; 2 SFB 1199, Leipzig University, Nikolaistr. 6-10, 04109 Leipzig, Germany

**Keywords:** Agri-food tech, Imaginaries, Financialization, Digitization, Venture capital, Technology

## Abstract

Over the past decade, investments in agricultural and food technology startups have grown to previously unknown dimensions. Mushrooming agri-food tech startups that promise to solve critical issues in the agri-food system through technological innovation are increasingly perceived as an attractive new investment opportunity for venture capitalists and investors. This paper investigates how digital agri-food technologies are narrated, constructed, and promoted for financial investment. Through qualitative content analysis of agri-food tech industry reports, articles, and commentaries we trace the logic, rationales, and narratives of this most recent investment rush, and reveal its immanent techno-finance fixes. We conceptualize the agri-food imaginaries produced within the agri-food tech discourse as *financialized* imaginaries, and argue that they are specifically tailored to construct, incentivize, and legitimize this new agri-food tech space for financial investment. In their attempt to raise money from investors, venture capital firms further fuel this development by discursively creating an ‘agri-food tech investment rush’—similar to the land and gold rushes of the past. Investments in agri-food tech startups, however, are presented to investors as both a profitable investment opportunity as well as a moral obligation, allowing for food production to cope with neo-malthusian and environmental threats. This paper contributes to our understanding of digitization as a socio-technical project, which includes the active envisioning and promotion of desirable agri-food futures.

## Introduction

If we believe the recent agri-food tech talk, we are currently ‘on the cusp of a global food revolution’ (UBS 2019, p. 8). Agri-food tech entrepreneurs and venture capitalists are eager to let us know that in the near future virtually nothing about our food system will remain the same, as novel and innovative technologies disrupt every single aspect of how we produce, distribute, and consume food. When diving into the glossy reports of the agri-food tech industry, loaded with tables, graphs, and narratives about a more sustainable and prosperous food system, we learn that the agricultural industry ‘is about to be disrupted and will transform into a high-tech industry’ (Monitor Deloitte 2016, p. 4) with technological innovations changing ‘the very fundamentals of how, where, and when we grow food’ (UBS 2019, p. 39). The projected change ‘from agriculture to agtech’ is touted as no less than leading to a ‘millennial shift from family farms to smart “food factories”’ (Monitor Deloitte 2016, p. 4), which also means that the ‘food we eat and the beverages we drink today may not be the same as those tomorrow’ (UBS 2019, p. 14).

Indeed, over the past decade, the digitization of agri-food has picked up pace rapidly, and investments in new digital agricultural and food technology startups have grown to previously unknown dimensions. The annual investment in agri-food tech startups increased from US$ 2.9 billion in 2012 to US$ 26.1 billion in 2020 (AgFunder 2021). Agri-food tech startups are mushrooming and promise solutions to a host of critical issues in the food system, ranging from ‘climate change to plastic pollution to inhumane treatment of animals’ (Fairbairn and Guthman 2020, p. 587;﻿ see also Schneider 2018; Fairbairn et al. 2022). In their attempt to raise money from investors, agri-food tech startups and venture capital firms fuel this development by discursively creating an ‘agtech investment rush’, suggesting that the time for ‘disruption’ is now. Such investments are presented as both a profitable investment opportunity as well as a moral obligation, allowing for food production to cope with neo-malthusian and environmental threats. How can we make sense of this recent agri-food tech hype, and the agri-food narratives and imaginaries of food futures it produces?

To answer this question, this paper investigates how startup companies, venture capital investment firms, and tech entrepreneurs discursively construct new technology-driven agri-food imaginaries. Embedded within the broader ‘financial culture’ of venture capital, startups, and technological entrepreneurialism these agri-food imaginaries can be conceptualized as *financialized* imaginaries, as they present current agri-food challenges and their desirable food future counterparts in ways that specifically target financial investors. Following Eve Chiapello, we consider the construction of these agri-food imaginaries as part of ongoing financialization, understood ‘as a specific process of transforming the world, objects, organizations and the problems we encounter, by the introduction of “financialized” practices, theories and instruments’ (Chiapello 2020, p. 81). Advancing financialization includes particular discursive ways of representing the world, constructing problems, suggesting solutions, and creating expectations, which are grounded in, and speak to, financial economic logics and needs. These discursive constructions, we suggest, also entail and produce certain imaginaries of the world or, to borrow from Jasanoff’s notion of sociotechnical imaginaries, ‘visions of desirable futures’ (Jasanoff 2015, p. 322).^1^ The crucial role of imagination, narration, and storytelling in finance has been well established in social studies of finance and cultural economy approaches (e.g. Beckert and Bronk 2018; Komporozos-Athanasiou and Fotaki 2020; Tarim 2012; Tsing 2000; Vint 2019). Drawing on this scholarship, we hold that the imaginaries of food produced in the proliferating agri-food tech discourse are specifically tailored to construct, incentivize, and legitimize this new space for financial investment.

Following this observation, this paper makes three main points. First, we argue that while the exaggerated agri-food tech language of superlatives, disruptions, and revolutions is rather novel in agri-food, it is far from uncommon within the startup and venture capital scene. Rather, these rhetorical strategies are prominent features of startup and venture capital discourses, such as the cleantech or bio economy discourses (Goldstein 2018; Lafontaine et al. 2021; Hogarth 2017; Knuth 2017). The discursive construction of agri-food technologies follows familiar strategies of what Anna Tsing described as ‘conjuring capital’ (Tsing 2000). Faced with a lack of quantifiable facts—usually considered the ‘hard currency’ of financial investment—entrepreneurs and investors recourse to the art of narration and spectacle to create ‘affective experiences’ (Hellman 2020, p. 106) for investors, that appeal to their emotions and moral missions.

Secondly, while the emerging literature engaged with agri-food tech has notably pointed to its entrepreneurial visions (Schneider 2018), its inherent ‘solutionism’, ‘technological fixes’, and ‘technology treadmills’ (Fairbairn and Guthman 2020; Reisman 2021; Bronson and Sengers 2022), as well as ‘promissory narratives’ and ‘non-disruptive disruptions’ (Guthman and Biltekoff 2020; Sexton et al. 2019; Stephens and Ruivenkamp 2016), we emphasize the financial character underlying the agri-food tech discourse, which has remained underinvestigated (for a recent exception see Fairbairn et al. 2022).^2^ Startups and venture capital firms pursue financial business models focused on ‘pitching’ their growth potential and missions to investors. Startup and venture capital’s constructions of the world and their ways to identify problems, suggest solutions, and provide moral justifications, we argue, can only be adequately understood if their inherent financial logics and argumentative strategies are brought into focus. This paper points to the financial character of the mushrooming agri-food tech scene and identifies the food imaginaries it produces as inextricably intertwined with its particular financial culture. Rather than solely trying to technologically ‘fix’ the food system, the agri-food tech scene promotes a ‘techno-finance fix’ (Morgan 2018), where the food system is sought to be ‘fixed’ through both technological progress and financialized means of technology development and profit-making from these technologies.

Lastly, we contend that the recent ‘agri-food tech hype’ needs to be examined in the broader context of the financialization of agri-food. Stepping out of its traditional function of providing farmers and agriculture with capital, over the past decade finance has gained a new role in the agri-food system and has started to reshape the power relations between agri-food actors. From agricultural derivatives trading and index-based agricultural insurances to the construction of agriculture based asset classes and the promotion of new farmer subjectivities, financial logics and rationalities have started to alter global agri-food relationships in profound ways (e.g. Clapp 2014; Fairbairn 2014, 2020; Larder et al. 2018; Bjørkhaug et al. 2018; Ouma 2020a). To these areas of the financialization of agri-food, this paper adds the aspect of financialized imaginaries as a particular *financialized* way of both (re-)imagining and thereby bringing about food futures. Recognizing the agri-food imaginaries produced in the agri-food tech discourse as financialized imaginaries is important in order to take them for what they are—not real-life, ‘everyday’ scenarios of our food future but rhetorical means to attract investment for this new investment space.

With this focus, this paper contributes to our understanding of digitization as a socio-technical project, where digitization is discursively advanced via the production of powerful visions of the future and appears as the space of the ‘collective enunciation of a joint desire’ (Forney and Dwiartama, this issue). We explore the project character of the agri-food tech discourse specifically in regard to its interplay with Forney and Dwiartama’s notion of ‘everyday digitization’, which points to the domestication of, and everyday experience with, digital technologies. We argue that while the everyday is seemingly absent—and arguably even needs to be concealed—within the digitization project, the project itself entails as well as produces its very own, peculiar ‘everyday-ness’.

The remainder of this paper is organized as follows. The next section depicts the ‘financial cultures’ of startups and venture capital firms as crucial actors within the emerging agri-food tech scene. We then sketch out our methodology and give some background information on key actors we investigated as part of our discourse analysis. This is followed by a presentation of the narratives and imaginaries employed within the agri-food tech discourse in which food is produced *for* finance. We identify five key elements in this discourse: (1) the construction of a ‘problem’, namely the food system in crisis; and (2) its counterpart, the suggestion of a technologically driven ‘solution’; which is (3) simultaneously presented as an investment opportunity (‘techno-finance fix’); (4) reinforced by the creation of an ‘agri-food tech investment rush’; and combined with (5) moral incentives. We conclude by reflecting on the significance of these agri-food imaginaries, and what they tell us about the interplay between ‘project’ and ‘everyday’ digitization.

## Financial cultures of startups, venture capital, and tech entrepreneurialism

Finance can be understood as an industry that produces, collects, and distributes money (Ortiz 2020, p. 5). Being made up of different companies, institutions, and actors, who pursue multiple aims and operate across various jurisdictions, financial practices differ depending on the social setting. At the same time, finance represents a space of increasingly standardized procedures, methods, and forms of knowledge regarding the production, collection, and distribution of money, which have reached ‘relative uniformity’ across the industry (Ortiz 2020, pp. 5–6). Startups, venture capital, and tech entrepreneurialism occupy a distinct space—and form part of a particular ‘financial culture’—within the broader financial industry, which share broader principles of financial capitalism as well as exhibit their very own practices, norms, and moralities. Understanding the specific ‘financial culture’ of the emerging agriculture and food tech scene is critical to assess the imaginaries, visions, and solutions its actors are currently suggesting for the agri-food system. By speaking of the ‘financial culture’ we do not intend to reify the problematic and essentializing notion of ‘culture’. Rather, we use the notion of ‘financial culture’ here to point to a broader set of shared practices as well as a particular self-image and ethos shared by people and institutions involved in this space (Ho 2009, p. 6; see also Pryke and du Gay 2007; Zaloom 2006). These, we suggest, produce particular engagements with, and approaches to, food and food futures, which deserve critical examination. This section sketches out the business model of the startup and the investment models of venture capital and impact investment to identify some core elements of this financial culture.

Startups are companies in their initial or early business stages. They concentrate on the development of a single product or service they believe is of value or in demand, and which they seek to bring to market, but lack capital to do so (investopedia, nd). Startup entrepreneurs are thus focused on ‘pitching’ the growth potential of their product or service to different funders, such as venture capitalists, angel and impact investors, or other private individuals. This focus on rapid and scalable growth is a key feature of startups, which, in the words of startup ‘guru’ Steve Blank, makes a startup ‘a temporary organization designed to search for a repeatable and scalable business model’ (Ready 2012). Startups seek to generate ‘big returns’ for their investors by aiming at being bought by larger companies, becoming a public company via IPO (initial public offering), or direct stock exchange listing. Another key feature of startups is their ‘disruptive moral ambition of doing something new or novel, of solving a particular problem, and bringing about meaningful change: ‘A successful start up typically wants to solve a problem and make the world a better place’ (McGowan 2018).

English-Lueck, who studied the emerging Silicon Valley startup scene and its ‘ecosystem’ of incubators, accelerators, and pitches, described their ‘entrepreneurial cultures’ as revolving around ‘techno-optimism’ and ‘technolust’ (English-Lueck 2017, p. 98/39). In this community, she writes, ‘technology takes on a heroic role’, the future is seen through ‘a rose-colored technological lens’, and the production of technology is considered ‘a moral mission’ which even gains a degree of spirituality (English-Lueck 2017, p. 98/39). Social entrepreneurship, venture philanthropy, and humanitarian design are further important components of this scene, which she identifies as ‘manifestations of intentional social change’: ‘Actors in this realm are more deliberately “changing the world” by applying the processes of design thinking, rapid deployment, and careful monitoring and reinvention to social issues ranging from poverty to housing’ (English-Lueck 2017, p. 9).

The self-presentation of tech entrepreneurship as a neutral and meritocratic economic field, however, does not necessarily correspond with reality, as Kelman (2018) argues. She concludes that rising to the top of the startup ecosystem often requires financial and cultural capital so that the resources successful tech entrepreneurs were able to mobilize become a matter of socioeconomic privilege. In a similar vein, Pfeilstetter finds that the lofty rhetoric of sharing, community, or collaboration employed by startup and tech entrepreneurs is rather covering up market competition, self-interest, and its promotion by lobbyists, corporations and the state. Manchester’s startup community, he argues, works as a ‘discursive tool’ to effectively communicate business information and achieve publicity, attention, legitimacy, and success:

‘The habit of portraying any event as successful, the need to document success online through measuring and statistics, the real-estate business capitalizing on the image of the NQ [Manchester’s Northern Quarter] as an entrepreneurial place or the constant ambivalence between competitive self-promotion and unconstrained self-realization at events, show that the “startup community” is also a market place and an advertising platform’ (Pfeilstetter 2017, p. 12).

In addition to capital from private individuals (such as family or friends), the business model of startups is to attract investment from venture capitalists as well as, more recently, so called ‘impact’ and ‘angel’ investors. Venture capital is a form of financial capital specialized in providing capital to startups and other small companies considered as showing potential for exponential growth. This focus on exponential growth is part of venture capital’s portfolio strategy (Hellman 2020, p. 109). Usually, investments are spread across many startups with the hope that at least one company will show the enormous success that outweighs other mediocre investments or failures. This ‘hits business’ or ‘long tail distribution of pay-offs’ is what sets venture capital investment apart from other forms of investment (Nicholas 2019, p. 1). From its origins in investments by wealthy individuals venture capital is now undertaken by specialized firms and represents a large scale and impactful part of entrepreneurial finance (Nicholas 2019, p. 3). Venture capital firms raise capital and then channel it into startups on behalf of their investors (e.g. pension funds, university endowments, insurance companies; called ‘limited partners’). They thus intermediate between investors and startups, for which they receive an annual management fee and a ‘carried interest’ (a share of the profits generated) (Nicholas 2019, p. 2).

Venture capital’s financial strategy on rapidly growing risky startups combines with a particularly enthusiastic and optimistic language and ethos, as well as with features such as ‘speculative valuations’ and ‘gut judgements’ (Hellman 2020, p. 99). Based on his research on the nascent cleantech scene of New York City in the mid-2000s, Goldstein finds that:

‘Venture capitalists present themselves as adventuresome, risk-taking pioneers, operating at the cutting edge of technological innovation and driving the motor of progress. In the field of cleantech, this self-image becomes closely linked to technological salvation—by investing in the most innovative, most “disruptive” clean technologies, venture capitalists are working to reshape our world, to commercialize the technosocial foundations of a better, cleaner economy.’ (Goldstein 2018, p. 96).

Thus, in line with the ethos of the startup culture identified above, venture capitalists exhibit similar moral imperatives, as they seek to transform life on this planet and like to imagine themselves as ‘world-makers’ and ‘entrepreneurial spirits who want to make an impact-beyond-capital’ (Goldstein 2018, p. 96). However, he concludes, more often than not these ambitions and moral aspirations are abandoned due to the factual constraints posed by fiduciary responsibilities, which posit the maximization of profit as the first and undisputed priority.

Over the past two decades, these forms of ‘risky’ investments have further diversified with the emergence of ‘impact investing’ and so called ‘angel’ investors. Impact investing seeks ‘more than financial returns’ by having a measurable impact on specific public, social, or environmental issues (Chiapello and Knoll 2020; Langley 2020). Promising both social and financial gains, impact investors can be seen as representing ‘the financial face of philanthro-capitalism’ (Langley 2020, p. 337). Angel investors in turn invest at the early stages of a company and can be both conventional or impact investors. As opposed to venture capitalists, angel investors are wealthy individuals who are not professional financiers and invest their own money without any fiduciary obligations (Hellman 2020). Considered rather ‘unsophisticated’ by venture capital firms, this new group of investors ‘find [in startup investment] a wide-open field to engage in an exciting activity—to wield a bit of influence, offer advice, and get close to the apparent heartbeat of innovation’ (Hellman 2020, p. 101).

In recent years, a broad variety of both conventional and impact investors have started to engage with agri-food tech, and set up venture capital funds specifically dedicated to investments into startup companies emerging within this space. As this particular entrepreneurial and financial culture discovers agri-food, what kind of food narratives does it produce? How do venture capitalists and startup companies construct imaginaries of food and food futures for their investors? Below, after outlining our methodology, we will illustrate these questions based on our empirical material.

## Methodology

To critically analyze the agri-food tech discourse, this paper draws on investment and industry reports, self-presentations, and thought pieces produced by the leading actors of the agri-food tech investment space. We determined leadership by the actors’ visibility within and outside of this space. We examined reports and brochures of agri-food tech actors and promoters, analyzed the websites of startups and venture capital firms operating in the agri-food tech space, and closely followed the media coverage of the sector, including industry newsletters and reports, podcasts, and publicly available discussion rounds and interviews with investors and entrepreneurs. We also attended one major industry event as well as various ‘demo days’, webinars, and pitch sessions in which startups presented their business model to potential investors. Due to COVID-19 restrictions these events were held online and were open to the public. One key actor in the field is the venture capital firm AgFunder (agfunder.com). Silicon Valley based, AgFunder was founded in 2013 to invest specifically in agriculture and food technologies. With its annual investment reports on the industry, newsletters, and hosted events, the firm and its media outlet AgFunderNews is a major driver of the agri-food tech space, setting the tone for new trends as well as writing the sector’s history. Further examples are venture capital firms such as SVG Ventures, s2g ventures, or Finistere Ventures. We included these firms in our analysis because they are listed among the most active accelerator funds and venture capital fund managers in the sector determined by the number of companies they have invested in (AgFunder 2021). Additionally, we analyzed industry reports from major consultant companies (e.g. Monitor and Deloitte) and banks (e.g. UBS), which also report about investment opportunities and trends in the sector.

We used the qualitative data analysis software MaxQDA to code and analyze our data following critical discourse analysis (Fairclough 2010). Our analysis has further been informed by the insights gained during ongoing empirical research on the emerging agri-food tech industries in the Netherlands (Dolinga) and California (Sippel). With regard to geographical representations, it should be noted that our data largely stems from Global North contexts, with main actors being based in North America, Europe, or Israel. The agri-food tech imaginaries presented in this paper can thus more precisely be identified as agri-food tech imaginaries originating from the Global North. Even if Northern entrepreneurial and financial actors often perceive themselves and their visions as ‘global’ (Ho 2005), which also applies to the actors investigated here, important regional differences exist (Wahome and Graham 2020). Our focus on the Global North is, however, reflective of the geographical spread of the agri-food tech industry. Although there has been some geographical diversification in recent years (with China and Latin America becoming more prominent regions), in terms of investment activity the agri-food tech scene is still largely concentrated in the Global North, with the United States, and California in particular, dominating the sector (AgFunder 2021).

## Constructing food *for* finance

In this section, we demonstrate how the emerging agri-food tech scene narratively constructs food for finance. Five main rhetoric elements can be found in this discourse. First, there is the presentation of a ‘food system in crisis’, establishing the need for immediate action for food system change. We further identify a ‘techno’ and a ‘finance fix’ as solutions for this crisis, which, as ‘techno-finance fix’ combine technological solutions with financial investment opportunities. Fourth, we argue that there is an ‘agri-food tech investment rush’ reminiscent of previous ‘rushes’, such as the ‘land rush’ of 2010. Fifth, and lastly, we suggest that agri-food tech investments are bound up with a moral discourse of ‘doing well while doing good’.

### The problem: a food system in crisis

One of the key operations within financialization is what Chiapello (2020, p. 85) identifies as ‘problematization’, namely ‘operations through which things and activities are redefined as questions of investment’. Relying on discursive and ideological work, problematization includes the relabeling and conceptual reframing of issues (e.g. as investment, return, risks), and the establishment of something as a problem that needs to be solved. This is then followed by a suggestion of the appropriate type of ‘solution’ to this problem: ‘elements of the situations concerned must be presented as an investment problem, for example under-investment requiring support from financial investors’ (Chiapello 2020, p. 85). Problematization is a prominent theme within the agri-food tech discourse. Virtually every report, brochure, or webinar starts with an assessment of the ‘status quo’ of the food system to then come to the unanimous diagnosis that we are faced with a system in crisis. This diagnosis, as such, is shared by critical food scholars and activists. Tanja Schneider (2018), for instance, finds similarities between the agri-food tech discourse and the positions voiced by alternative food networks and NGOs in regard to environmental issues and questions of sustainability. The reasons identified for, and the solutions proposed to engage with, this crisis, however, differ substantially. In the agri-food tech discourse, the food crisis is usually grounded in the increasing demand for food due to a growing global population on the one hand and changing dietary patterns on the other:

‘The population is growing at approximately 77.6 million per year, and it is expected to reach nearly 10 billion by 2050. At the same time, the middle class is expected to double by 2030. And as incomes rise people spend more on food (Engel’s law) and eat more animal protein (8 pounds of grains are needed for 1 pound of beef). To meet the demand for food, fuel and fiber from a growing and increasingly affluent population, experts predict that we will need to double global crop production over the next 35 years’ (Leclerc and Tilney 2015).

Following Malthusian logic, the increase in food demand from a growing and more affluent world population is translated into a production challenge. These ‘food logics’—food is a necessity, population growth means food insecurity will continue to grow, and dietary habits are changing towards increasingly animal protein-based diets—were a common narrative used to rationalize and legitimize long-term investment in food production for investors in the land rush discourse (Fairbairn 2014; Larder et al. 2015). The difference in the agri-food tech discourse is that only the fact that ‘people need to eat’ does not turn food into a good investment as increasing productivity is in itself presented as problematic: ‘We are approaching the tipping point. As the population continues to grow, the food systems [are] becoming less viable for consumers and the planet’ (AgriNovus Indiana 2019, March 15). The current food system is seen as unsustainable and inefficient to a degree that expanding it further will come at the price of ‘potentially devastating costs’:

‘The current agricultural business model has to take some responsibility for the untenable state of affairs today. In their quest to produce ever more food, farmers have been incentivized to disregard environmental costs, leading to a depletion of biodiversity, pollinators (e.g. bees) and soil health, as well as the social costs associated with scarce resources like water and energy’ (UBS 2019, p. 11).

As ‘we are nearing the world’s natural limits’ (UBS 2019, p. 11), increasing food production to the requested levels is simply not possible: current agricultural systems are unsustainable and inefficient; the advances from the Green Revolution and mechanization are exhausted; and rates of yield production are trending negatively (e.g. CPT Capital, n.d.). Sustainability pressures, environmental degradation, scarce resources, and climate change are all mentioned as further negatively impacting food production. Moreover, climate change induced water scarcities, pests, and diseases are predicted to result in considerable productivity loss—but unfortunately, current solutions are ‘either toxic to people and the environment or ineffective. So there really has to be something done’ (AgFunder 2020a, August 8). These inefficiencies of the current food system are not limited to food production alone. Also food waste is identified as a ‘major economic and environmental issue’ (UBS 2019, p. 28). Lastly, the pandemic is presented as having shone a light on the critical issues our food system is facing, from disruptions of global supply chains and logistics networks to labor issues, tons of food being wasted to increasing food insecurity and food nationalism (S2G 2020a, p. 2). In short, as AgFunder’s Co-Founder Michal Dean puts it in an online investor webinar: ‘It’s a really, really bad situation at the moment and things are going to get worse’ (AgFunder 2020a, August 8).

Given the urgency and planetary scale of the problem, how can our food system be ‘saved’? As mentioned above, problematization, as an important part of financialization, also includes the identification of an appropriate ‘solution’ to the problem. These solutions need to be made ‘tangible’ by giving ‘embodiment’ to the suggested visions. ‘Tangibilization’ usually involves the production of knowledge and expertise about the new area of investment, such as its qualities and risks, as well as some form of quantification, such as figures and models that lend ‘credibility to the theory that they are worthy of investment’ (Chiapello 2020, p. 86). Problematization and tangibilization work closely together:

‘Thanks to problematization, special narratives using the language of investment and its returns, of capital and its risks, make thinkable the possibility of attracting financial actors and persuading them to finance what is under-invested. Thanks to the tangibilization work, it is then possible to identify what is worth investment, assign values, and incorporate them into calculations’ (Chiapello 2020, p. 86).

How does tangibilization work, however, in a business where there is little yet to quantify, and where the main selling point is the proclamation of future potential, the creation of an expectation? Faced with this challenge, startups and venture capital resort to what Anna Tsing (2000) calls the ‘self-conscious making of a spectacle’ as part of the ‘economy of appearances’: they ‘dramatize their dreams in order to attract the capital they need to operate and expand’ (Tsing 2000, p. 118). Tangibilization as well as spectacular dramatization and exaggeration are common features of the agri-food tech discourse. Below, we first illustrate how the ‘spectacle’ is initiated by imaginaries of technologically enabled ‘bright and shiny’ food futures as a classical example of a ‘techno fix’, that is to say the positioning of technologies as solutions to social problems (Morozov 2013). We then demonstrate that this techno fix is only one side of the coin, as these techno-food futures are simultaneously touted as an investment opportunity, leading to what we identify as a ‘techno-finance-fix’.

### The techno-fix: techno-food future imaginaries

In speculative enterprises, writes Tsing, profit must be imagined before it can be extracted: ‘the possibility of economic performance must be conjured like a spirit to draw an audience of potential investors’ (Tsing 2000, p. 118). Tsing uses the term ‘conjuring’—‘to call forth spirits and to perform magical tricks’—to characterize the specific features of entrepreneurial strategies where everyday performance requirements are turned into ‘dramatic shows of potential’. The term emphasizes the intentionality of the performance, which, by making use of the charisma of the performer, hopes to move the audience beyond the limits of rational calculation: ‘Conjuring is supposed to call up a world more dreamlike and sweeter than anything that exists; magic, rather than strict description, calls capital’ (Tsing 2000, p. 120). We suggest that the first step of ‘conjuring capital’ in the agri-food tech discourse is the depiction of technologically driven and enabled food futures that are described as safer, healthier, and more sustainable than any food system we have known so far. As much as the portrayal of our current agri-food system is one of crisis, thanks to ingenious entrepreneurs who are not shying away from ‘moonshots’ in envisioning ‘out of the box’ technological innovations, the future of food—as agri-food tech envisions it—is full of hope and promise.

This food system of the future, we are promised, is a ‘nutritious and accessible food system for everyone’ (AgFunder 2019, December 5), built on the principles of healthiness and humaneness (Stray Dog Capital, n.d.), as well as ‘sustainable production, equitable distribution, and healthy consumption’ (Cultivian Sandbox, n.d.). This food system is also of ‘greater sustainability, security and safety’ (Cultivian Sandbox, n.d.) and provides ‘healthy nutritious food for all’ while ‘conserv[ing] our planet’s precious resources for future generations’ (UBS 2019, p. 6). It will be a ‘more innovative and resilient future food system’ (S2G Ventures 2020a) that provides ‘affordable nutrients for 10 billion people, preserves and regenerates natural resources, actively contributes to decarbonization and protects land and ocean biodiversity’ (Astanor Ventures, n.d.).

This food future will be brought about by fusing physical, digital, and biological technologies: ‘Only highly disruptive, scalable new technology guided by a deep understanding of, and respect for, nature will revolutionize the agrifood sector’ (Astanor Ventures, n.d.). ‘Widespread adoption of precision technology’ will allow for farming to become ‘truly automated’ and ‘factory-like with robots on the ground as well as in the air’ (UBS 2019, p. 33). Farming will increasingly take place in controlled environments, with leafy greens, herbs, and other fruit and vegetable innovations being grown in vertical indoor or container formats (S2G Ventures 2020b). Evidently, also the food of the future will look entirely different from today. Meat might come from the laboratory instead of the country (UBS 2019, p. 14) and supermarket shelves will be filled with plant-based alternatives to dairy and ‘palatable all-vegan egg’ (Leclerc and Tilney 2015).

However, such substantial change will not be achieved with only one innovation or product—what is needed is rather a full makeover of the system (AgriNovus Indiana 2019, March 15). As the entire food system model has to be remade from a ‘resource-intensive one into a fully sustainable one’ (UBS 2019, p. 5), the key actors identified as capable of achieving this fundamental change are entrepreneurs whose innovations will be ‘fundamental to addressing the problems and finding solutions for farmers, consumers, and the environment’ (THRIVE 2020, p. 5). It is thus the ‘intersection of technology and entrepreneurship’ that will define the future of food and agriculture (S2G Ventures 2020a, p. 2: 1701) and through which ‘agrifood can be transformed from one of the leading causes of social and environmental harm into the greatest regenerative solution’ (Astanor Ventures 2021, p. 3).

In short, in the agri-food tech investment discourse we find a host of imaginaries of food futures that are ‘more dreamlike and sweeter than anything that exists’ (Tsing 2000, p. 120) in the world of agriculture and food. In this imaginary food future, the issues the current food system is facing have been overcome through the combination of innovative technologies and entrepreneurialism. These techno-food future imaginaries entail sweeping narratives of solutionism and ‘technological fixes’, where technological innovations are presented as solutions to social and ecological crises, promising new eras of prosperity, freedom, and equality (e.g. Huesemann and Huesemann 2011; Johnston 2018; Katzenbach 2021). This techno-fix, the strong faith that technology adaption and entrepreneurialism will act as ‘saviors’, as necessary and functional solutions for social, political, and cultural problems, represents a major narrative throughout the agri-food tech discourse. The techno-fix is, however, only one component of a rhetoric strategy. It further combines with a strong belief in financial logics and instruments as the best way to achieve these futures, resulting in what Morgan identifies as a ‘techno-finance fix’ (Morgan 2018). The imaginary food futures described above are only one element within the ‘conjuring of capital’. They are complemented with additional ‘tangibilization’ work—the creation of convincing narratives about investment opportunities to attract the necessary capital for their implementation.

### Techno-finance fixes: investment opportunities

Given the urgency and planetary scale of the problem, how can our food system be brought into this ‘clean’ and ‘sustainable’ future? How can the development of technologies be afforded? As Morgan (2018, p. 9) writes, ‘over-hubristic faith in technological solutions is not without its connections to power and capital’. In the agri-food tech discourse, it is precisely the problem—‘the food system is in crisis’—which makes agri-food ripe for those disruptions that offer huge investment opportunities. The dramatic spectacle of agri-food tech investment, we argue, includes an additional layer, where, in the transition from crisis to future, skyrocketing possibilities for profit making are lying idle for investors. In the agri-food tech discourse we can thus identify a ‘techno-finance fix’, which combines the dominance of a strong faith in technologies and technological determinism of the techno fix described above with a financial approach for addressing these issues via the means and instruments of the increasingly financialized global economy. These two are not separate processes but rather in a ‘symbiotic and sometimes dialectical relationship operating under the conditions of global capital’, and ‘[i]n their mutual enforcing of each other [they] combine to a powerful narrative’ (Morgan 2018, p. 9).

The ‘finance fix’ unfolds as follows. In the early years of agri-food tech development around 2010, agri-food tech was described as an ‘underinvested space’, which was just being discovered by investors. AgFunder, for instance, described agri-food as ‘massively undercapitalized’ as investors were disinterested and ‘didn’t see it as a sexy, viable space’ (AgFunder 2020b, May 12). This, in 2013, led to the founding of AgFunder, which was grounded in the assumption that a better food and agriculture system was ‘being held back by both a lack of investment and ultimately investor literacy’ (AgFunder 2020b, May 12). Similarly, Aaron Rudberg, managing director of the venture capital firm S2G, stated at the 2018 Agbioscience Innovation Summit that ‘with so much disruption, everyone must be investing’. To his disappointment, however, food and agriculture had long remained an underinvested sector relative to GDP: ‘The food and ag industry is one of the largest in the world. Yet it doesn’t receive the [equivalent] amount of venture capital’ (AgriNovus Indiana 2019, March 15). In the mid-2010s, however, it was claimed this situation was starting to change with more investors coming to the agtech investment space, ‘particularly the large corporates’ (AgriNovus Indiana 2019, March 15). Also Rob Leclerc and Melissa Tilney from AgFunder stated in 2015, ‘[i]t’s taken a while, but investors and entrepreneurs have started to take notice’ as agri-food tech was surpassing sectors such as fintech or cleantech (Leclerc and Tilney 2015). Investments, it was claimed, were now on ‘an exponential growth path’, which was even ‘surprising traditional and long-established players’ (Monitor Deloitte 2016, p. 5).

More recently, the sector has been presented as having matured to a certain extent, as AgFunder found in 2021: ‘It was exciting to see earlier-stage companies raise larger rounds than the first wave of innovators’ (AgFunder 2021, p. 8). At the same time, the venture capital firm is quick at pointing out that this should not be interpreted as a sign of slow down or less opportunities being available. In fact, quite the opposite: ‘With talent moving from first wave companies to second, we expect the sector to accelerate rapidly’ (AgFunder 2021, p. 8). In other words, agri-food tech is currently no longer presented as a ‘niche, experimental and risky sector’. Instead, median deal-size growth is seen as signaling ‘maturity of first wave innovation’ (AgFunder 2021, p. 8).

These assessments of the recently increasing investment activities are further combined with the prospect of massive but still unexplored future market opportunities. For instance, it is claimed that ‘[t]he need for agri-food tech innovation is greater than ever’, which has created many opportunities for entrepreneurs and technologists (AgFunder 2021, p. 54). Further, as UBS explains, until recently, ‘agriculture lagged all other industries in terms of disruption’. Now, however, ‘this figure is set to spike’: ‘Given the vast, untapped market and the rapid emergence of powerful technologies, we expect food innovation to become a USD 700bn market by 2030—a fivefold jump from today’ (UBS 2019, p. 7). Lastly, there is reason for optimism, as ag and food tech are ‘part of a huge industry [w]orth close to US$9 trillion, which is about 10% of global GDP’ (AgFunder 2020b, May 12).

In short, in the early years of the nascent agri-food tech investment space, the finance fix consisted of a self-presentation of the sector as ‘under-invested’ and hence lucrative. With increasing investment activity recorded, the observation that investments were taking off was then presented as giving reason to expect even greater opportunities in the future. In the ‘finance fix’, there is thus a fine balance between providing evidence that ‘others have already invested’ in order to increase the credibility and maturation of the sector on the one hand, while convincing investors that many investment opportunities have not been exploited yet on the other.

The presentation of investment opportunities is further rhetorically rationalized by two major and broader societal trends, the ‘fundamentals’ that prove the case of agri-food tech investments. First, a change in consumer preference is identified as a ‘principal disruptive force’ of agri-food system change (UBS 2019, p. 5). Consumers are described as becoming more concerned about the state of our food system and its environmental impact, and increasingly demanding healthy, sustainable, local, socially conscious, and environmentally friendly foods (AgFunder 2020a, b, May 12). Meeting these new and thus far unmet consumer demands is presented as a massive opportunity for investors as they cannot be achieved through the traditional methods of the current food system.

A second major driver of food system transformation identified is a greater societal shift towards sustainability, which, again, makes agri-food tech a good investment opportunity as it matches both a societal trend and need. The importance of sustainability is also more and more recognized by investors making the agri-food sector an attractive addition to investors’ portfolios:

‘Institutional investors have increasingly adopted sustainability as a key performance indicator for their portfolio companies. They’re starting to see the application of ESG (environmental, social and governance principles) as a key driver of operational efficiency, sustainability, and, perhaps in the current climate and most importantly, resiliency. [L]arge investors are seeing climate risk as a fundamental threat to their portfolios, and therefore their investors’ money. [I]mpact capital is beginning to flow into food and agriculture like never before’ (AgFunder 2020a, August 8).

While both changing consumer behaviors and societal values are translated into massive, yet unmet demands, technological advancements make these novel and innovative solutions possible—while simultaneously offering possibilities for profit making themselves. Lastly, COVID-19 is presented as further reinforcing these two trends (S2G Ventures 2020a) as it ‘highlighted the importance of efficient supply chains and alternative ways of growing, processing, transporting and selling food to consumers’ (AgFunder 2021, p. 3). The pandemic year of 2020 has been called ‘a blow-out year’ for agri-food tech as startups raised another record amount of capital (AgFunder 2021, p. 3).

### The time for investment is now: the agri-food tech rush

Picking up on the argument of the ‘economy of appearances’, the ‘spectacle’ is often accompanied by an additional temporal component, which renders the scenario of the spectacle even more pressing and urgent. As Li (2014, p. 596) points out, dramatic shifts or narratives are used to create ‘investment rushes’, alerting investors to the temporal fugaciousness of the investment opportunity and trying to convince them of the ‘why now?!’. This investment rush, she writes, usually consists of establishing ‘a sudden, hyped interest in a resource because of its newly enhanced value, and the spectacular riches it promises to investors who get into the business early. Hence the rush. Do it now before others spot the value, and the profit margins decrease’ (Li 2014, p. 595).

As in the land rush of 2010, the construction of a ‘rush’—the effort to provide a compelling argument as to why the time for investment is now—is another prominent feature of the agri-food tech discourse. We frequently find claims suggesting that the ‘fourth agricultural revolution is already on its way and you should invest now’ (Deloitte 2016, p. 5), that ‘there is no better time to invest in food and agriculture than now’ (UBS 2019, p. 5), or that ‘now is the time to invest in agri food tech’ (AgFunder 2021, p. 3). As AgFunder’s Yanniv Dorone explains in a webinar:

‘Now, as venture capitalists, the questions that we need to ask [are]: is there an investable opportunity here? Is this something that is going to end here? Or is it going to turn into an even bigger direction? And so what we realize is that *right now, there is a perfect storm* that [m]akes this market a very interesting investment opportunity’ (AgFunder 2019, December 5; own emphasis).

**Fig. 1:**
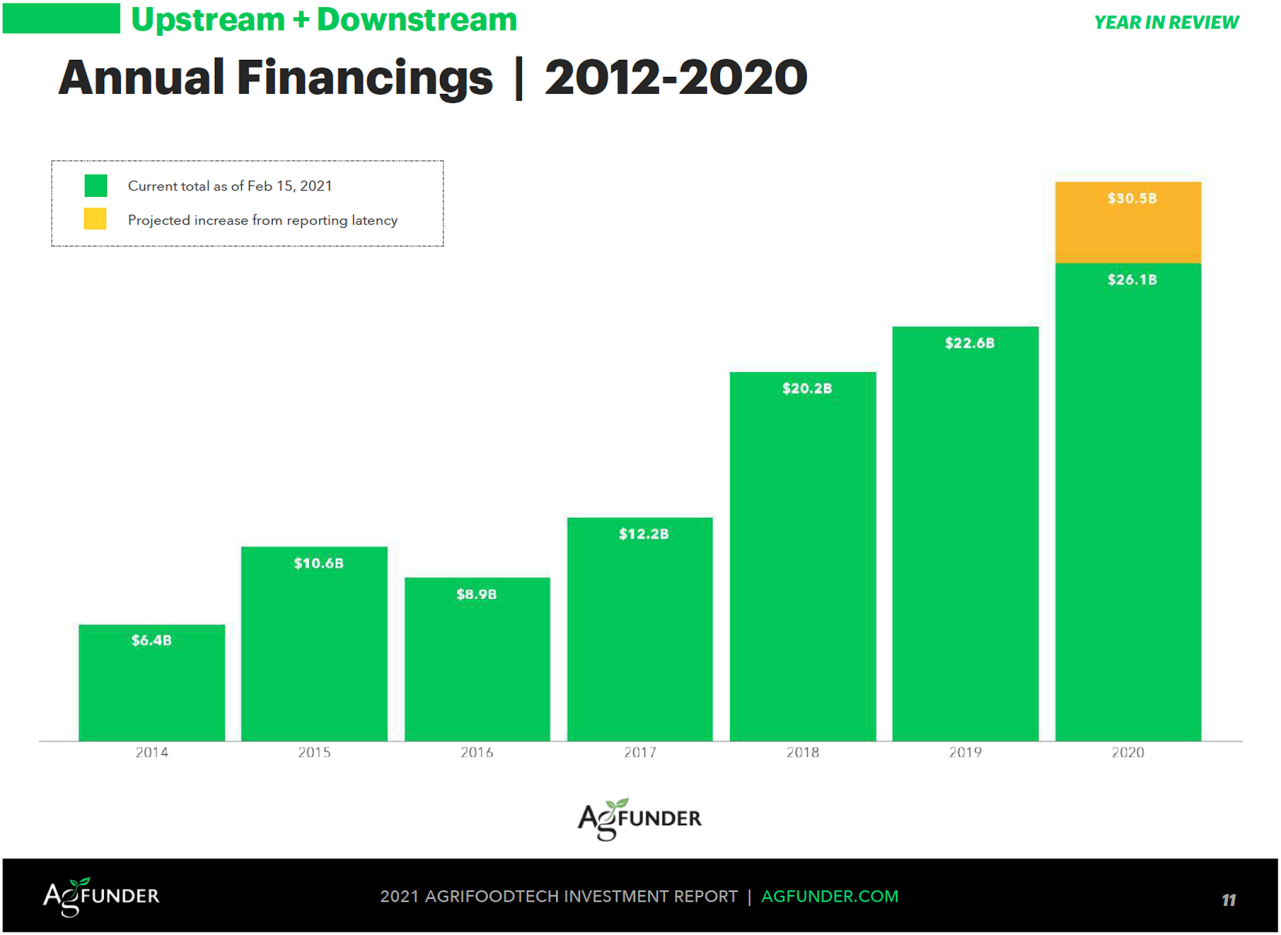
Visual illustration of agri-food tech investment growth (2012–2020) (AgFunder 2021, p. 11)

**Fig. 2:**
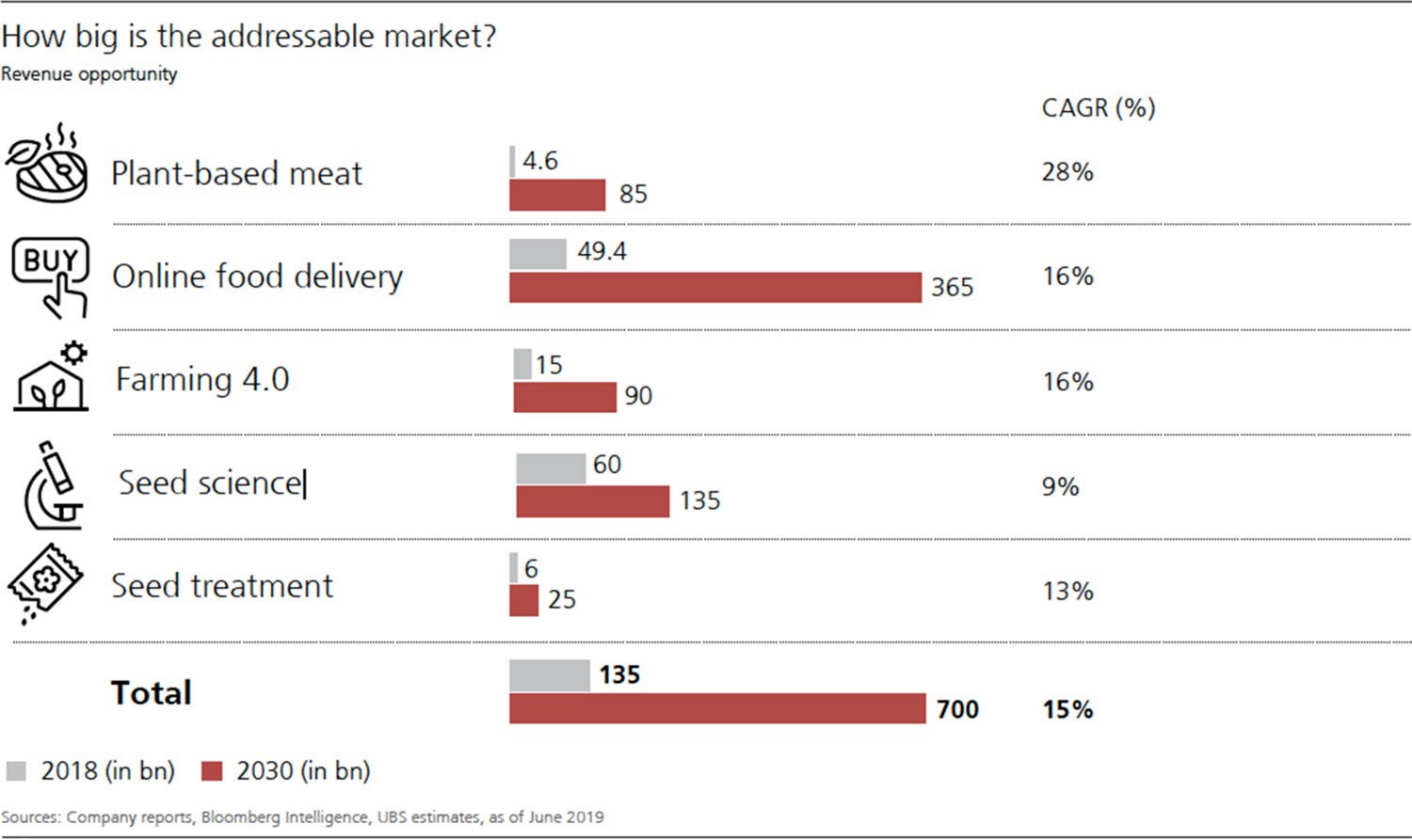
Visual illustration of investment opportunity (UBS 2019, p. 67)

All components of the discourse analyzed so far—the problematization, the spectacle of imaginary techno-food futures, the ‘techno-finance fix’, and the agri-food tech investment rush—are discursively produced and performed during conferences and webinars, as well as made visually ‘tangible’ via the presentation of facts, figures, and graphs in reports, presentations, newsletters, and on websites. In all these settings, claims are rhetorically supported by using a language of ‘disruption’, ‘revolution’, and ‘exceptionalism’, combined with what could be considered a variant of ‘statistical picturing’ (Demeritt 2001). Here, the credibility and authority of visual representations, such as graphics, diagrams, or tables, is used to provide ‘evidence’ for both a certain argument, as well as the necessity or profitability of specific actions, in this case financial investment. In AgFunder’s annual investment reports, for example, the well-known ‘hockey stick’ graphs function as a visual means to demonstrate the significance of the growth of the agri-food technology sector (see Fig. 1). Similarly, the UBS report uses graphic strategies to ‘depict’ estimated agri-food tech investment opportunities, thereby making these opportunities visually perceptible and ‘real’ for potential investors (see Fig. 2).

### Doing good by doing food: the moral impetus of agri-food tech investment

Lastly, an important feature of the agri-food tech investment discourse is its moral claim of providing ‘investment with purpose’. This moral impetus is another parallel between the agri-food tech discourse and the land rush discourse, with similarities and differences in how morality is being claimed for the respective investment contexts. As mentioned above, underlying ‘food logics’ serve as legitimizing narratives in both discourses. Investments in land were often morally justified by pointing to their necessary contribution to counter future food insecurity in the light of population growth, changing dietary habits, and the surge of biofuel production. These underlying themes are equally present in the agri-food tech discourse, where we can find a similar narrative of agri-food investments fulfilling ‘basic needs’:

‘Our team has made this our life’s work because, you know, what we’re doing is both meaningful and impactful. And (agri-)food is interesting because it really sits at the base of Maslow’s hierarchy of needs. And when you can strengthen that foundation, you get leverage to move economies and make societies more resilient’ (AgFunder 2020b, August 8).

What sets the agri-food tech scene apart from the land investment community is its more prominent claim of being in the game for both ‘mission *and* profit’. While the ‘mission’ component has not been entirely absent in the land rush discourse, the land rush discourse has mostly been dominated by the moral narratives of the ‘mainstream investors’ identified by Kish and Fairbairn (2018, p. 571), for who producing economic value serves as a basis for moral claims-making (see also Larder et al. 2015; Ouma 2020b; Sippel 2018). In agri-food tech, the moral purpose of bringing about the better ‘food futures’ depicted above—and thereby helping to solve environmental issues, address climate change, and bring about a more sustainable food system—appears as a paramount ‘mission’ of agri-food tech investment also in the mainstream discourse. Here, this mission and the necessity of economic profitability are flawlessly blending: ‘From a mission perspective, building a sustainable food system is the right thing to do. From an investment perspective, we believe it will continue to deliver profitable returns’ (S2G Ventures 2020a, p. 2). ‘Doing good by doing food’, the slogan of the Israeli incubator The Kitchen FoodTech Hub (2021, February 18), or AgFunder’s aspiration ‘to solve big problems with big market potential’ (Leclerc and Tilney 2015) further illustrate this unification of purpose and profit. What is more, the ‘mission’ component is claimed to make the difference between current investments in the sector and its historical predecessors:

‘Historically, innovations like precision agriculture were developed by businesses purely for commercial reasons. But this time we can expect to see significant growth in the scale and impact of social enterprises on all aspects of daily life, fuelled by entrepreneurs with a desire to not just make money but to also make a difference by investing for good’ (UBS 2019, p. 26).

In short, reflecting the financial culture of startups and venture capital outlined above, the agri-food tech discourse exhibits a strong morality where doing good and doing well are not seen as opposing goals but rather smoothly go hand in hand. There are no trade-offs between return and impact as the more successful the investment, the more ‘moral impact’ is to be expected.

## Conclusion

The mushrooming agriculture and food tech scene has recently received increasing attention in agri-food studies. This paper has pointed to the financial component of the emerging agri-food tech scene, and argued that it produces discourses and imaginaries of food *for* finance. Rather than being just one element of this emerging scene, we suggest that the financial character is located at the heart of the agri-food tech endeavor, making its analysis crucial for the understanding of these most recent developments in agri-food. In other words, addressing the ‘financial culture’ of startups and venture capital is key to understanding why the proponents of the agri-food tech discourse construct food in certain ways, and to adequately assessing the food future imaginaries resulting from this discourse. Rather than being novel or unique, we demonstrate that this discourse employs specific narratives and follows rhetoric patterns that are quite common within the financial culture of startups and venture capital. This, we have shown, includes a particular, and often linear way of storytelling that starts with the construction of a ‘problem’ to which techno-financial ‘solutions’ are then being promoted, followed by the creation of temporal urgency and moral justifications. While some actors, such as impact investors, more strongly emphasize the moral components within their discourse, we could find elements of this core rhetorical figure with every actor we investigated in our analysis. A crucial component within this discourse is what we identified as a ‘techno-finance fix’, pointing to the prevalence of faith in technological innovation and progress combined with the use of financial rationales as appropriate means to facilitate and catalyze the ‘fixing’ of sustainable food future challenges. Within this logic, financial capital is reframed from being a cause of social and economic issues to now being ‘part of the solution’ (Chiapello and Knoll 2020, p. 17). The particular imaginaries of techno-food futures that the agri-food tech scene envisions need to be interpreted as fulfilling a dedicated function within the ‘conjuring of capital’ for this new field of technology development.

We want to conclude by pointing out two insights resulting from our findings for the ambivalent interplay between digitization as a ‘project’ and the notion of ‘everyday’ digitization (Forney and Dwiartama, this issue). First, our paper illuminates the ambiguity between project and everyday digitization as mutually relying on, as well as concealing one another. Within the agri-food discourse we investigated in this paper, the everyday, the mundane, the messy, and the uncontrolled aspects of digital food futures are largely absent. Nowhere in our data did we learn about the ‘everyday’ of agri-food tech, be it its concrete applications, regional differences, challenges within specific situations, or farm level details. The agri-food discourse sits at the ‘macro’ level, its playing field is the globe, its aspirations are big and universal—it does not know of nor require any concrete locations, materialities, or fixations. This, we suggest, is no coincidence, as the everyday struggles, setbacks, and failures arguably need to be concealed within this discourse to make the project of digitization work. Even more so, the complications of the everyday are not only a hindrance to the digitization project, but especially to its financial driving force. The discursive construction of the digitization project lives off the illusion it creates that hides and obscures the messiness of the everyday as much as any other hint of uncertainty or lack of control.

Second, if the food future imaginaries we identified in this paper are indeed constructed for finance, what does this mean for our everyday imaginaries of the future of food, and the role that digitization can play within this? How should we engage with the food future imaginaries produced by the agri-food tech scene, which have arguably already started to capture our collective imaginations of how we envision the future of agri-food? Studying imaginaries allows us to become aware of the role and purpose that imaginaries play within specific societal projects. Imaginaries are never neutral but closely intertwined with particular environmental or societal undertakings and endeavors, as underlying implicit understandings or explicit drivers of envisioned futures (Sippel and Visser 2021). If the imaginaries of food and food futures produced in the agri-food tech discourse are indeed *financialized* imaginaries, as we suggest in this paper, their main objective is to attract capital to this new field of technology development. Financialized imaginaries, in other words, are geared towards envisioning future worlds in ways that open them up to, and make them accessible to, financial investment and its logics and return generating mechanisms. If such imaginaries become part of our ‘everyday imagination’ of food futures, we need to be careful to not misconstrue them as ‘realistic’ depictions of our future world as their function first and foremost remains to raise capital, and not to provide us with real-life, ‘everyday’ scenarios of what our food future will look like. Financialized imaginaries are part of the digitization project and not of its everyday realization, and thus should not be mistaken as such. By highlighting the possibilities, necessities, and constraints that the financial culture of venture capital and startups forces on the discourse of the digital agricultural ‘revolution’ (Rose, this issue), this paper has emphasized the significant gap between the promises of digitization as a revolutionary and disruptive project and the realities that ‘everyday digitization’ holds for food and agriculture today as well as in the future.

## Footnotes

1 We draw on Jasanoff, who defines ‘sociotechnical imaginaries’ as ‘collectively held, institutionally stabilized, and publicly performed visions of desirable futures’ (Jasanoff 2015, p. 322). While the imaginaries of food futures identified in this paper are publicly performed, their institutionalization is arguably still in the making. The ‘collective’ that holds these imaginaries might at this point mostly be restricted to the members of the broader agri-food tech scene.

2 In their discourse analysis of precision agriculture (PA) discourses more generally, Duncan et al. (2021, p. 1187) recently came to a similar conclusion, namely that ‘PA is re-inventing itself to attract investment for new devices that promise revolutionary advances in farming’.

## References

[CR1] AgFunder. 2019, December 5. Investing in Alternative Protein Startups, with AgFunder [Video file]. YouTube. https://www.youtube.com/watch?v=ZPZddeNK_Cc. Accessed September 7 2021.

[CR2] AgFunder. 2020a, August 8. AgFunder GROW Impact Fund Webinar [Video file]. YouTube. https://www.youtube.com/watch?v=TSHfrgrMC4I. Accessed September 7 2021.

[CR3] AgFunder. 2020b, May 12. Invest in a more robust, resilient food system, with AgFunder’s Fund III (second close webinar) [Video file]. YouTube. https://www.youtube.com/watch?v=fNldOHZeYDQ. Accessed September 7 2021.

[CR4] AgFunder. 2021. AgFunder AgriFoodTech Investment Report. https://agfunder.com/research/. Accessed September 7 2021.

[CR5] AgriNovus Indiana. 2019, March 15. Investing in Tomorrow’s Food + Ag | 2018 Agbioscience Summit [Video file]. YouTube. https://www.youtube.com/watch?v=LWIds6ef698. Accessed September 7 2021.

[CR6] Astanor Ventures. 2021. Astanor Ventures Impact Creation Report 2020–2021. https://astanor.com/wp-content/uploads/2021/10/Astanor-Ventures-Impact-Creation-Report-2021.pdf. Accessed May 9 2022.

[CR7] Astanor Ventures. n.d. Our Priorities. https://astanorprod.wpengine.com/about/. Accessed May 9 2022.

[CR8] Beckert, J., and Bronk, R. 2018. An Introduction to Uncertain Futures, in (ibid.) (eds) *Uncertain Futures: Imaginaries, Narratives, and Calculation in the Economy*. Oxford: Oxford University Press, 1–38.

[CR9] Bjørkhaug H, Magnan A, Lawrence G (2018). The Financialization of Agri-food Systems.

[CR10] Bronson K, Sengers P (2022). Big tech meets big ag: Diversifying epistemologies of data and power. Science as Culture.

[CR11] Chiapello, E., and Knoll, L. 2020. Social finance and impact investing. Governing welfare in the era of financialization. *Historical Social Research/Historische Sozialforschung*, 45 (3): 7–30.

[CR12] Chiapello E, Mader P, Mertens D, van der Zwan N (2020). Financialization as a Socio-technical Process. The Routledge International Handbook of Financialization.

[CR13] Clapp J (2014). Financialization, distance and global food politics. The Journal of Peasant Studies.

[CR14] Cultivian Sandbox. n.d. We help build next-generation food and agriculture technology companies. https://cultiviansbx.com. Accessed September 7 2021.

[CR15] CPT Capital. n.d. About. https://cptcap.com/about/. Accessed May 9 2022a.

[CR16] Demeritt D (2001). Scientific forest conservation and the statistical picturing of nature’s limits in the Progressive-era United States. Environment and Planning D: Society and Space.

[CR17] Duncan E, Glaros A, Ross DZ, Nost E (2021). New but for whom? Discourses of innovation in precision agriculture. Agriculture and Human Values.

[CR18] English-Lueck J (2017). Cultures@SiliconValley.

[CR19] Fairbairn M, Guthman J (2020). Agri-food tech discovers silver linings in the pandemic. Agriculture and Human Values.

[CR20] Fairbairn M (2014). “Like Gold with Yield”: Evolving Intersections between Farmland and Finance. The Journal of Peasant Studies.

[CR21] Fairbairn M (2020). Fields of Gold: Financing the Global Land Rush.

[CR22] Fairbairn, M., Kish, Z., and Guthman, J. (2022) Pitching agri-food tech: Performativity and non-disruptive disruption in Silicon Valley, *Journal of Cultural Economy* (online 22 Jun 2022).

[CR23] Fairclough N (2010). Critical Discourse Analysis: The Critical Study of Language.

[CR24] Forney, J., and Dwiartama, A. (forthcoming) The project, the everyday, and reflexivity in sociotechnical agri-food assemblages: proposing a conceptual model of digitalization. Forthcoming in *Agriculture and Human Values.* 10.1007/s10460-022-10385-4 PMC10241715 37287886

[CR25] Goldstein J (2018). Planetary Improvement: Cleantech Entrepreneurship and the Contradictions of Green Capitalism.

[CR26] Guthman J, Biltekoff C (2020). Magical Disruption? Alternative Protein and the Promise of de-Materialization. Environment and Planning E: Nature and Space.

[CR27] Hellman J (2020). Feeling good and financing impact. Historical Social Research/historische Sozialforschung.

[CR28] Ho K (2005). Situating global capitalisms: A view from wall street investment banks. Cultural Anthropology.

[CR29] Ho K (2009). Liquidated. An ethnography of wall street.

[CR30] Hogarth S (2017). Valley of the unicorns: Consumer genomics, venture capital and digital disruption. New Genetics and Society.

[CR31] Huesemann M, Huesemann J (2011). Techno-fix: Why technology won’t save us or the environment.

[CR32] Investopedia, n.d. https://www.investopedia.com/terms/s/startup.asp. Accessed September 3 2021.

[CR33] Jasanoff S, Jasanoff S, Kim S-H (2015). Imagined and invented worlds. Dreamscapes of Modernity: Sociotechnical Imaginaries and the Fabrication of Power.

[CR34] Johnston SF (2018). The technological fix as social cure-all: Origins and implications. IEEE Technology and Society Magazine.

[CR35] Katzenbach C (2021). “AI will fix this”—The technical, discursive, and political turn to AI in governing communication. Big Data and Society.

[CR36] Kelman S (2018). The Bumipreneur dilemma and Malaysia’s technology start-up ecosystem. Economic Anthropology.

[CR37] Kish Z, Fairbairn M (2018). Investing for profit, investing for impact: Moral performances in agricultural investment projects. Environment and Planning a: Economy and Space.

[CR38] Knuth S (2017). Green devaluation: Disruption, divestment, and decommodification for a green economy. Capitalism Nature Socialism.

[CR39] Komporozos-Athanasiou A, Fotaki M (2020). The imaginary constitution of financial crises. The Sociological Review.

[CR40] Lafontaine C, Wolfe M, Gagné J, Abergel E (2021). Bioprinting as a sociotechnical project: imaginaries, promises and futures. Science as Culture.

[CR41] Langley P (2020). Assets and assetization in financialized capitalism. Review of International Political Economy.

[CR42] Larder N, Sippel SR, Lawrence G (2015). Finance capital, food security narratives and Australian agricultural land. Journal of Agrarian Change.

[CR43] Larder N, Sippel SR, Argent N (2018). The redefined role of finance in Australian agriculture. Australian Geographer.

[CR44] Leclerc, R., and Tilney, M. 2015. AgTech Is The New Queen Of Green. TechCrunch. http://tcrn.ch/1HjwtoY. Accessed September 7 2021.

[CR45] Li TM (2014). What is land? Assembling a resource for global investment. Transactions of the Institute of British Geographers.

[CR46] McGowan, E. 2018. What Is a Startup Company, Anyway? What is a startup? Let these startup founders clear it up once and for all. (March 1, 2018) https://www.startups.com/library/expert-advice/what-is-a-startup-company. Accessed September 3 2021.

[CR47] Monitor Deloitte. 2016. From Agriculture to AgTech. An industry transformed beyond molecules and chemicals. https://www2.deloitte.com/content/dam/Deloitte/de/Documents/consumer-industrial-products/Deloitte-Tranformation-from-Agriculture-to-AgTech-2016.pdf. Accessed September 7 2021.

[CR48] Morgan T (2018). The techno-finance fix: A critical analysis of international and regional environmental policy documents and their implications for planning. Progress in Planning.

[CR49] Morozov E (2013). To Save Everything, Click Here: Technology, Solutionism, and the Urge to Fix Problems That Don’t Exist.

[CR50] Nicholas T (2019). VC. An American History.

[CR51] Ortiz H (2020). A political anthropology of finance: Studying the distribution of money in the financial industry as a political process. Anthropological Theory.

[CR52] Ouma S (2020). Farming as a Financial Asset: Global Finance and the Making of Institutional Landscapes.

[CR53] Ouma S (2020). This can(’t) be an asset class: The world of money management, “society”, and the contested morality of farmland investments. Environment and Planning a: Economy and Space.

[CR54] Pfeilstetter R (2017). Startup communities: Notes on the sociality of tech-entrepreneurs in Manchester. Journal of Comparative Research in Anthropology and Sociology.

[CR55] Pryke M, du Gay P (2007). Take an Issue: Cultural Economy and Finance. Economy and Society.

[CR56] Ready, K. 2012. A Startup Conversation with Steve Blank. (Aug 28, 2012) https://www.forbes.com/sites/kevinready/2012/08/28/a-startup-conversation-with-steve-blank/. Accessed 3 September 2021.

[CR57] Reisman E (2021). Sanitizing agri-food tech: COVID-19 and the politics of expectation. The Journal of Peasant Studies.

[CR58] Rose, D. et al. (forthcoming) The old, the new, or the old made new? Everyday counter-narratives of the so-called fourth agricultural revolution. Forthcoming in *Agriculture and Human Values*. 10.1007/s10460-022-10374-7 PMC9628410 36340284

[CR59] S2G Ventures. 2020a. Everyone Eats—The Future of Food in the Age of COVID-19. Why innovation and entrepreneurship are more important than ever. https://www.s2gventures.com/reports/everyone-eats---the-future-of-food-in-the-age-of-covid. Accessed September 7 2021.

[CR60] S2G Ventures. 2020b. Growing Beyond the Hype. Controlled Environment Agriculture. https://www.s2gventures.com/reports/growing-beyond-the-hype%3A--controlled-environment-agriculture. Accessed September 7 2019.

[CR61] Schneider, T. 2018. Promising sustainable foods. Entrepreneurial visions of sustainable food futures. Phillipov, Michelle and Katherine Kirkwood (eds) *Alternative Food Politics: From the Margins to the Mainstream*, Routledge: London, 75–94.

[CR62] Sexton AE, Garnett T, Lorimer J (2019). Framing the future of food: The contested promises of alternative proteins. Environment and Planning E: Nature and Space.

[CR63] Sippel SR (2018). Financialising farming as a moral imperative? Renegotiating the legitimacy of land investments in Australia. Environment and Planning a: Economy and Space.

[CR64] Sippel SR, Visser O (2021). Introduction to symposium “Reimagining land: Materiality, affect and the uneven trajectories of land transformation”. Agriculture and Human Values.

[CR65] Stephens N, Ruivenkamp M (2016). Promise and ontological ambiguity in the in vitro meat imagescape: From laboratory myotubes to the cultured burger. Science as Culture.

[CR66] Stray Dog Capital. n.d. About Us. https://straydogcapital.com. Accessed May 9 2022b.

[CR67] Tarim E (2012). Storytelling and structural incoherence in financial markets. Journal of Interdisciplinary Economics.

[CR68] The Kitchen FoodTech Hub By Strauss. 2021, February 18. What’s Cooking? The Kitchen Demo Day 2021 [Video file]. YouTube. https://www.youtube.com/watch?v=LPw_1cLRI-Y. Accessed September 7 2021.

[CR69] Thrive by SVG Ventures. 2020. Agtech Thrive Top 50. 50 Growth Stage Companies Disrupting the Future of Food and Agriculture. https://thriveagrifood.com/startups/thrive-top-50/. Accessed September 7 2021.

[CR70] Tsing A (2000). Inside the economy of appearances. Public Culture.

[CR71] UBS. 2019. The food revolution. The future of food and the challenges we face. https://www.ubs.com/global/en/wealth-management/chief-investment-office/sustainable-investing/2019/food-revolution.html. Accessed September 7 2021.

[CR72] Vint, S. 2019. Promissory Futures: Reality and Imagination in Finance and Fiction. *CR: The New Centennial Review* 19(1): 11–36.

[CR73] Wahome M, Graham M (2020). Spatially Shaped Imaginaries of the Digital Economy. Information, Communication and Society.

[CR74] Zaloom C (2006). Out of the Pits: Traders and Technology from Chicago to London.

